# Pulsed electromagnetic energy treatment offers no clinical benefit in reducing the pain of knee osteoarthritis: a systematic review

**DOI:** 10.1186/1471-2474-7-51

**Published:** 2006-06-15

**Authors:** Christopher James McCarthy, Michael James Callaghan, Jacqueline Anne Oldham

**Affiliations:** 1 Warwick Emergency Care and Rehabilitation, Warwick Medical School, University of Warwick, Coventry, CV4 7AL, UK; 2 The Centre for Rehabilitation Science, University of Manchester, Manchester, M13 9WL, UK

## Abstract

**Background:**

The rehabilitation of knee osteoarthritis often includes electrotherapeutic modalities as well as advice and exercise. One commonly used modality is pulsed electromagnetic field therapy (PEMF). PEMF uses electro magnetically generated fields to promote tissue repair and healing rates. Its equivocal benefit over placebo treatment has been previously suggested however recently a number of randomised controlled trials have been published that have allowed a systematic review to be conducted.

**Methods:**

A systematic review of the literature from 1966 to 2005 was undertaken. Relevant computerised bibliographic databases were searched and papers reviewed independently by two reviewers for quality using validated criteria for assessment. The key outcomes of pain and functional disability were analysed with weighted and standardised mean differences being calculated.

**Results:**

Five randomised controlled trials comparing PEMF with placebo were identified. The weighted mean differences of the five papers for improvement in pain and function, were small and their 95% confidence intervals included the null.

**Conclusion:**

This systematic review provides further evidence that PEMF has little value in the management of knee osteoarthritis. There appears to be clear evidence for the recommendation that PEMF does not significantly reduce the pain of knee osteoarthritis.

## Background

Osteoarthritis is a major health problem affecting over 60% of adults in the Western world over 65 years of age, yet it receives scant resources for treatment or clinical research [1,2]. The knee is one of the most commonly affected joints and patients present with a combination of pain, deformity, inflammation, stiffness and muscle atrophy. With the lack of cure, none surgical treatment is usually directed to symptomatic relief and prevention of functional dysfunctio [3]. One such treatment modality is pulsed electromagnetic field therapy, that uses electromagnetic fields with an on-off effect of pulsing to produce athermal effects that promote tissue healing and relieve pain and inflammation [4]. Despite the lack of knowledge of its effects over placebo, this modality has increased in use significantly over the last 25 years [4].

There has been some analysis of the data regarding the effectiveness of pulsed electromagnetic fields (PEMF) with knee osteoarthritis patients. Marks et al [3] published a literature review on the effects on knee osteoarthritis of both pulsed and continuous shortwave diathermy. The equivocal results of this review were attributed, in part, to the poor methodological quality of the 11 non-randomised comparative and randomised controlled trials (RCTs) they studied. Hulme et al. [5] published a Cochrane review, in 2001. They identified only 3 papers, of adequate quality for review, totalling 259 patients, and concluded there was a lack of evidence of a clinically important effect of PEMF in the treatment of knee osteoarthritis.

We have become aware of several prospective randomised controlled trials comparing PEMF to placebo PEMF, in the treatment of knee osteoarthritis. Thus, we undertook a systematic review of the studies published in the last 10 years to specifically evaluate PEMF for patients with knee osteoarthritis.

## Methods

We performed a similar search strategy to that used by Hulme et al 2001 [5] with the following criteria for inclusion in the review.

### Types of studies

1. Randomised controlled trials

2. Controlled clinical trials

Trials were not included if they measured bone and cartilage repair from electromagnetic therapy following a specific treatment for osteoarthritis, which would then no longer be applicable to all knee OA patients.

### Types of participants

Those trials with subjects over 18 years of age, with clinical and radiological confirmation of the diagnosis were considered.

### Types of intervention

All types of PEMF and Pulsed Electrical Stimulation trials were included. Although the latter relies on direct application of an electrical field rather than creating induced current through magnetic impulses, they act by the same mechanism. Trials that compared the intervention group using PEMF to a standard treatment were included, as well as placebo-controlled studies.

### Outcomes

The main outcomes of interest were pain and functional disability as recorded by validated self report instruments such as the Western Ontario and McMasters University Osteoarthritis Index WOMAC [6], EuroQol [7], Arthritis Impact Measurement Scale AIMS[8] or SF-36 [9]. Studies that did not utilise validated outcome measures of pain and function were excluded.

We searched MEDLINE, AMED, EMBASE, HealthSTAR, CINAHL, PEDro, and SPORTDiscus and the Cochrane Controlled Trials Register (CCTR) from January 1966 to September 2005. The electronic search was complemented by the following hand searches of bibliographic references and abstracts published in special issues of specialized journals or in Conference Proceedings. Reference lists were hand-search for further identification of published work, presentations at scientific meetings and personal communications. Abstracts were not used if additional data could not be obtained. The publication bias could not be assessed due to the small number of included studies.

#### Search strategy

1 exp osteoarthritis/or osteoarthritis.tw.

2 electromagnetics.mp. or electromagnetic fields/[mp = title, abstract, registry number word, mesh subject heading]

3 electromagnetic$.tw.

4 exp electric stimulation therapy/

5 electrical stimulation.tw.

6 or/2–5

7 1 and 6

### Assessing quality

Abstracts were read to make the final decision on selection of the full paper for review. The quality of the papers was assessed by two reviewers independently using a validated criteria checklist[10]. The maximum total score achievable was 5. Any differences were resolved by discussion between the reviewers. Differences unresolved in this manner were resolved in discussion with a third reviewer.

### Statistical analysis

For pain and functional outcomes, weighted mean differences and standardised mean differences, with 95% confidence intervals, were calculated. Due to differences in follow up assessment timings, data were analysed from the immediate post-intervention assessments. A priori it was decided that an effect size difference in post treatment scores of greater than 0.2 was required to represent a clinically important difference. Effect sizes were calculated by dividing the difference in post-treatment group mean scores by the pooled standard deviation of both baseline scores.

## Results

The literature searches produced 20 randomised controlled trials and 5 non randomised clinical trials. Abstracts were viewed to exclude duplicate publications, narrative reviews, letters, commentaries, use of electrical stimulation that was not PEMF, pathologies other than knee osteoarthritis and non-randomised trials. Three studies, reported in previous systematic reviews were excluded due to their use of non-validated outcome measures [11-13]. As a result, five papers were found that met the review selection criteria. These were all RCTs with placebo controlled PEMF. All these trials used both a pain scale and a measure of self reported functional status. The five trials provided data for a total of 276 patients with knee osteoarthritis.

Two studies used low frequency PEMF ranging from 3 to 50 Hz requiring long durations of treatment (3 to 10 hours a week) [14,15] whilst three studies used typical "pulsed short wave", high frequency devices with shorter treatment durations [16-18]. The low frequency, long duration treatments showed a greater trend for short-term improvement in WOMAC function score than the high frequency, low duration treatments but an equivocal lack of benefit in pain relief.

**Figure 1:**
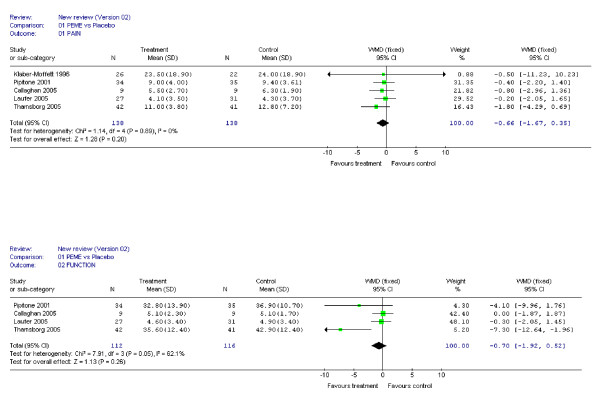
Plots of weighted mean difference for pain and functional disability. PEMF versus placebo PEMF (95% confidence intervals).

The highest score for study quality was 5/5 [19] with the lowest being 3/5 [15]. See Table 1. The weighted mean differences for improvement in pain and function were extremely small and well below the levels one might consider clinically significant. The weighted mean confidence intervals included the null value, for both outcomes, pain 0.66 (95%CI 0.35 to -1.67), function 0.70 (0.52 to -1.92) See Figure 1. Effect sizes were not statistically or clinically significant for all outcomes with the exception of function in one study, with a low quality score (3/5), (effect size for Function 0.59, 95%CI 0.14 to 1.12) [15].

## Discussion

It is unusual for a rehabilitation electrotherapeutic modality to be evaluated in a series of RCTs and thus to find a number of randomised controlled trials of intervention versus control was encouraging. The findings of this review support and update the work of previous reviewers [5]. Despite relatively low total patient numbers (n = 276) and some heterogeneity in the frequency of the PEMF used, it would appear that PEMF has little clinical value in reducing the pain of knee osteoarthritis. The assertion that PEMF has no clinically significant benefit, above that achieved with placebo treatment, on the pain of patients with knee osteoarthritis can be confidently proposed. An improvement in WOMAC physical function score was described by one study, however this study had a low methodological score and used a unique PEMF device, unreplicated by other studies. The same study did not report an improvement in pain [15].

## Conclusion

Current evidence would suggest that PEMF is unlikely to be a valuable contributor to multimodal rehabilitation of knee osteoarthritis. The resources currently being used for the provision of PEMF may be better utilised in the deployment of more clinically efficacious rehabilitation, such as in the provision of exercise classes and advice sessions [20, 21].

## Pre-publication history

The pre-publication history for this paper can be accessed here:



## Figures and Tables

**Table 1:** A table showing the methodological score, numbers, duration of treatment, outcomes measures and standardised mean difference for pain and function

Author	Quality Score[10] (0–5)	Number receiving PEMF	Number receiving placebo	Duration of Treatment (weeks)	Pain Outcome	Functional Outcome	Standardised Mean Difference for pain (95%CI)	Standardised Mean Difference for function (95%CI)
Klaber-Moffett et al 1996[16]	5	26	22	3	VAS Pain Scale	None reported	-0.03 (-0.59 to 0.54)	None reported
Pipetone et al 2001[14]	5	34	35	6	WOMAC Pain Scale	WOMAC Physical Function Scale	-0.10 (-0.58 to 0.37)	-0.33 (-0.80 to 0.15)
Thamsborg et al 2005[15]	3	42	41	6	WOMAC Pain scale	WOMAC Physical Function Scale	-0.31 (-0.74 to 0.12)	-0.58 (-1.02 to -0.14)
Laufer et al 2005[17]	3	27	31	3	WOMAC Pain scale	WOMAC Physical Function Scale	-0.05 (-0.57 to 0.46)	-0.09 (-0.60 to 0.43)
Callaghan et al 2005[18]	4	9	9	2	VAS Pain Scale	AIMS	-0.33 (-1.26 to 0.61)	0.00 (-0.92 to 0.92)
TOTAL		138	138				-0.16 (-0.39 to 0.08)	-0.33 (-0.59 to -0.07)

VAS – Visual analogue scale,

WOMAC – Western Ontario and McMaster's Universities OA index

AIMS – Arthritis Impact Measurement Scale
